# Sexual Attraction to Both Genders in Ambiphilic Men: Evidence from Implicit Cognitions

**DOI:** 10.1007/s10508-019-01552-6

**Published:** 2019-11-05

**Authors:** Robert J. Snowden, Ellen Fitton, Aimee McKinnon, Nicola S. Gray

**Affiliations:** 1 School of Psychology, Cardiff University, Cardiff, CF10 3AT UK; 2 Department of Psychology, Swansea University, Swansea, UK; 3 Abertawe Bro-Morgannwg University Health Board, Bridgend, UK

**Keywords:** Bisexual, Ambiphilia, Gynephilia, Androphilia, Implicit Association Test, Sexual orientation

## Abstract

Ambiphilic (or bisexual) men describe feelings of sexual attraction to both men and women. However, physiological measures of arousal have failed to show a consistent pattern of arousal to both genders. We measured men’s automatic associations between the concept of sex (represented by words) and the concepts of men versus women (represented by images) via the Implicit Association Test (IAT) and a priming task. On the IAT, gynephilic men (*N* = 32) were faster for women-sex pairings, androphilic men (*N* = 18) were faster for men-sex pairings, while ambiphilic men (*N* = 20) showed no bias toward either gender. We then isolated the concepts of “men” and “women” by comparing them separately against neutral images. In contrast to both the gynephilic or androphilic men, ambiphilic men showed sexual associations to both men and women. On the priming task, ambiphilic men showed faster responses to sex words, but slower responses to not-sex words, when primed with pictures of either men or women compared to when primed by neutral images. The results from all the experimental tasks suggest that ambiphilic men have a pattern of sexual association that is different from both gynephilic and androphilic men and represents a sexual attraction to both men and women.

**Electronic supplementary material:**

The online version of this article (10.1007/s10508-019-01552-6) contains supplementary material, which is available to authorized users.

## Introduction

The National Health Interview Survey (Ward, Dahlhamer, Galinsky, & Joestl, 2014) estimated that 0.7% of Americans classified themselves as bisexual or ambiphilic (sexually attracted to both men and women). Gates (2011) estimated that around 1.8% of the U.S. population identify themselves as ambiphilic (1.4% for men and 2.2% for women), while Copen, Chandra, and Febo-Vazquez (2016) report higher figures (2.0% and 5.5%, respectively). This higher reported prevalence is perhaps due to Copen et al. (2016) using a survey technique of computer-assisted self-interviewing where the person did not have to report their sexuality explicitly to an interviewer. If so, this may imply the presence of implicit biases against ambiphilia even in those individuals who report this form of sexual interest. In the UK as a whole, and also in Wales (the site of the current study), the figures appear similar with around 0.7% of people reporting being ambiphilic, with two-thirds of these being women (Welsh Government, 2017). Such figures suggest that the prevalence of ambiphilia is similar to that of gay/lesbians (gynephilic women or androphilic men, otherwise termed gay or lesbian men and women). However, ambiphilia has remained “hidden” in comparison (MacDowall, 2009) and individuals appear to suffer greater and unique forms of prejudice from many sources including “academicians and scholars, activists in lesbian and gay communities, and the popular press” (Brewster & Moradi, 2010). In part, such prejudices may arise from the idea that people reporting ambiphilia are “confused, experimenting, or in denial about their true sexual orientation” (Giaba, 2017). As such, research into ambiphilia is needed to counter such prejudicial thoughts and to examine the pattern of sexual attraction in ambiphilic individuals.

Previous research has noted that there can be discrepancies between a person’s self-reported sexual attractions (or, indeed, their self-reported sexual identity) and measures of sexual responses to stimuli. For instance, Chivers, Rieger, Latty, and Bailey (2004) found that both androphilic (attracted to men) and gynephilic (attracted to women) women showed strong genital responses to images of men and to images of women, while men showed a highly category-specific response with genital arousal being limited to images of their explicitly stated preferred gender. Hence, different methodologies of examining sexual attractions are likely to measure different stages of the psychological processes behind sexual attraction. While ambiphilic men clearly exist in term of their identity and behavior, it is less clear whether all aspects of their sexual attraction would show strong responses to both men and women given the previous findings that men tend to show category-specific responses.

Sexual arousal can be viewed as a process that is determined by many component features (Dewitte, 2016). According to the information processing model (Janssen, Everaerd, Spiering, & Janssen, 2000), the presentation of a stimulus leads to an automatic sexual appraisal that gives the stimulus emotional meaning via the matching of the stimuli in memory. It is also thought that this automatic process leads to genital responses if a sexual meaning is evoked. A second stage, also automatic, involves response generation that integrates this emotional meaning with response or motor plans which lead to the subjective experience of sexual arousal and also further genital response. These first two automatic stages can also trigger controlled attentional processing of relevant sexual information and activate the explicit meaning for the individual and the subjective experience of arousal (see also the emotion-motivation model of Dewitte, 2016). Hence, different features of the sexual response, such as genital arousal versus self-reported arousal, may be under the primary control of different systems and need not necessarily be in tandem. To understand sexual interest, we need measures that tap into different parts of these processes, and it may well be these different stages can produce different patterns of results.

Perhaps the most obvious measure of sexual attraction is the response of the genitals to sexual stimuli (Freund, 1963). Gynephilic (i.e., sexually attracted to women) men show substantially greater genital arousal when viewing sexual images of women than images of men, whereas androphilic men (i.e., sexually attracted to men) show the opposite pattern of results (Freund, Langevin, Cibiri, & Zajac, 1973; McConaghy, 1967). If ambiphilic men are attracted to both genders, then we should expect to see substantial genital arousal to images of men and to images of women. The results of such experiments have, however, not provided unequivocal evidence for this position. Tollison, Adams, and Tollison (1979) and Rieger, Chivers, and Bailey (2005) showed that the genital responses of ambiphilic men were similar to that of androphilic men in showing arousal to images of men and not to images of women. Cerny and Janssen (2011) also found that ambiphilic men showed a greater genital response than gynephilic and androphilic men to a “bisexual” stimulus that depicted a man engaged in sex with both a man and a woman. However, interpretation of these results is not straight forward. The finding of differences between the responses of ambiphilic men to the bisexual stimulus compared to gynephilic and androphilic men shows that they are in some way unique in their responses, but the study failed to find that the ambiphilic group had substantial genital responses to both images of men and to images of women (see Bailey, Rieger, & Rosenthal, 2011). Rosenthal, Sylva, Safron, and Bailey (2011) revised this issue with the idea that previous studies may have included some men in the ambiphilic group who might really be androphilic but would report some gynephilia in order to appear more “normal” and/or socially acceptable. They also suggest that some androphilic men might identify as ambiphilic in order to appear more masculine and increase their sexual appeal to other androphilic individuals. Rosenthal et al. used stringent criteria for participant inclusion in their experiments (such as having been in a sexual relationship with at least two partners of each sex). Participants watched videos of either two men having sex or two women having sex. Their data showed clear evidence that ambiphilic men show substantial genital arousal to both types of video, whereas gynephilic and androphilic men only showed substantial genital arousal to the video depicting their self-reported sexual preference.

A second well-used measure of sexual interest is the pupillary response (Hess, Seltzer, & Shlien, 1965). Rieger and Savin-Williams (2012) presented participants with videos of individuals that were masturbating. Ambiphilic men showed pupil dilation (the pupil becoming larger) when viewing either men alone masturbating or women alone masturbating, whereas gynephilic men only showed pupil dilation to the women and androphilic men only to the men (see also Rieger et al., 2015).

Hence, there is some evidence from both genital responses and from pupillary responses that ambiphilic men show arousal to stimuli that depict either men or women. While such results would appear to support that notion of a clear sexual response to both genders in ambiphilic men, there are some problems. First, the stimuli used are of a quite long duration (seconds or minutes) and clearly allow for the conscious appraisal of the stimuli. As such they are not good at informing about early, automatic evaluations of the stimuli. It is notable that some experiments that have used far briefer stimuli have suggested there may be problems in using pupillometry for this purpose. For example, Aboyoun and Dabbs (1998) found that there was greater dilation to nude compared to clothed images, and in particular to nude images of men, irrespective of the gender of the viewer. They suggest that this may be due to a “novelty” effect rather than sexual interest. Similar results have been recently reported (Snowden, McKinnon, Fitoussi, & Gray, 2019).

A third method(s) to examine sexual attractions is based on measures of cognitive processes. In order to assess these early automatic appraisals of the sexual stimuli, there are a range of indirect methods that have been developed and several of these have been used in the context of sexual attraction (e.g., viewing times: Imhoff et al., 2010; Stroop interference, Ó Ciardha & Gormley, 2013; implicit relational assessment: Timmins, Barnes-Holmes, & Cullen, 2016; choice reaction times: Wright & Adams, 1994; rapid serial visual presentation: Zappala et al., 2013; gaze patterns: Dawson, Fretz, & Chivers, 2017; dot-probe tasks: Snowden, Curl, Jobbins, Lavington, & Gray, 2016). However, only viewing time measures have been previously used to examine automatic appraisals of sexual stimuli in ambiphilic men. Both Ebsworth and Lalumière (2012) and Lippa (2013) show that ambiphilic men showed approximately equal viewing times to images of adult men and to adult women, whereas gynephilic and androphilic men spent much longer viewing their self-reported preferred gender. While these studies are supportive of a bisexual interest in ambiphilic men, it is not clear what psychological processes underpin the viewing time task (see Imhoff et al., 2010) and the long viewing time in these studies may well mean that the measures contain elements of reflective/deliberate/controlled processes rather than automatic appraisals of these stimuli.

In this paper, we have used two techniques, the Implicit Association Test (IAT: Greenwald, McGhee, & Schwartz, 1998) and the priming technique (Fazio, Sanbonmatsu, Powell, & Kardes, 1986), to examine these “implicit” sexual associations to images of men and women. The two techniques are related, but act at different levels of cognitive representation (see below).

### The Implicit Association Test

The IAT attempts to measure a person’s automatic associations between mental representations, such as sex and gender. Snowden, Wichter, and Gray (2008) developed an IAT where people classified words as sex-related or not-sex-related, and pictures as either men or women. In one condition, the pictures of men and sex words were paired by requiring the same response button (with the pictures of women and non-sex words being paired by a different response button). In the other condition, women and sex words were paired, etc. As expected, gynephilic men had good performance when women and sex were paired, while androphilic showed the opposite pattern of results. These results have been consistently replicated (Ciani & Battaglia, 2014; MacInnis & Hodson, 2013; Ó Ciardha & Gormley, 2013; Snowden, Craig, & Gray, 2011). Hence, this gender-sex IAT appears to be able to measure sexual appraisal of these briefly presented stimuli.

The gender-sex IAT has not previously been used to measure automatic sexual appraisals in ambiphilic men. Our hypothesis was that ambiphilic men would show sexual associations to both the images of men and women and this might result in a “null” result as these two associations would cancel. Note that ambiphilia does not demand equal attraction to both men and women, only that there is substantial attraction to both genders (the “minimum difference” concept—see Rieger et al., 2005) but that they are more attracted to men than gynephilic men, and more attracted to women than androphilic men. This was our prediction.

One of the limitations of the standard IAT is that it involves a comparison between two categories—in this case men and women. Some researchers have used a “single-item IAT” where only one concept (e.g., women) is presented (Karpinski & Steinman, 2006). However, this technique has problems due to lower reliability than the traditional IAT measure (Schnabel, Asendorpf, & Greenwald, 2008). A second technique is to replace one of the categories with a “neutral” stimulus. That was the technique used in the present study where we replaced the pictures of women with neutral pictures to produce a men-sex IAT and replaced the pictures of men with neutral pictures to produce a women-sex IAT. We predicted that both androphilic and ambiphilic men would perform better in the men-sex condition compared to the neutral-sex condition for the men-sex IAT, while there would be no effect for gynephilic men. For the women-sex IAT, we predicted that both gynephilic and ambiphilic men would perform better in the women-sex condition than the neutral-sex condition, while there would be no effect for androphilic men.

### Priming Task

In a sequential priming task, a stimulus (e.g., a picture of a woman) is presented and quickly followed by a target word that the person must classify (e.g., a sex or a not-sex word). Typically, the priming stimulus alters the ability of the participant to classify the target word—a priming effect (Cameron, Brown-Iannuzzi, & Payne, 2012). Crucially, the priming task does not require the person to classify the priming stimulus, and is thought to access automatic processes relating to the presentation of the individual priming exemplars (i.e., the automatic cognitions about the stimulus). The mechanisms of priming are still debated although consensus suggests that there are two possible mechanisms (Klauer & Musch, 2003). The first is that the stimulus causes automatic associations within the brain network that allow faster access to a particular target type—hence, the picture of a woman could lower the time needed to access the concept of sex for gynephiles. The second is that the stimulus primes a response and so a gynephile may be more ready to press the “sex” response button when primed with a picture of a woman. For the purposes of the present experiment, the mechanism of priming is irrelevant—both theoretical models are dependent on the prime producing a reaction that should be dependent on a person’s automatic evaluation of the prime.

This technique has only rarely been used to look at sexual associations (Snowden et al., 2008; Snowden & Gray, 2013). Snowden et al. (2008) showed that the technique was able to distinguish gynephilic and androphilic men with a high degree of accuracy; however, we know of no study to date that has examined the responses of ambiphilic men. We developed a priming task using the same stimuli as used for the IAT: the primes were pictures of men, women and neutral objects, and the same sex and not-sex words served as the targets. We predicted that gynephilic men would show faster RTs to sex words when the prime was a picture of a woman (rather than a man or neutral picture), and that androphilic men would show faster RTs to the sex words when the prime was the picture of a man (rather than a woman or a neutral picture). We hypothesized that the ambiphilic men would show faster RTs to sex words after both picture primes of men and of women in contrast to the neutral picture primes. For not-sex words, we predicted that all these patterns of results would be reversed.

## Method

All procedures for these experiments were given ethical permission from the Ethical Committee of the School of Psychology, Cardiff University.

### Participants

Participants were recruited from a range of advertisements using Facebook and Twitter. We also handed out leaflets and recruited participants from various events including BiFest Wales, PrideCymru mardi gras, and the LGBT + Society of Cardiff University. We encouraged participants to inform their friends about the experiment. We did not advertise for one or more particular group of people or sexual interest, but stressed that we were interested in human sexuality and that we wished to test people of all sexual interests. The leaflets/advertisements asked for participants willing to take part in our experiments. They stated that the experiments would involve images of a sexual nature and we would be asking them about their sexual interests and behaviors. People who agreed to be contacted gave contact details. They were then contacted to arrange a time to be tested. In all, 73 men were successfully recruited through this method. Due to the limited number of people of minority sexual interests and the difficulty in recruiting to such sensitive experiments, we determined that we would recruit at least 20 participants per group (gynephilic, ambiphilic, androphilic) which would allow us to detect a large effect size (*d *= 0.80) with conventional power (1 − beta = 0.80) and alpha (0.05) for a comparison of groups (*t* test). However, our adverts asked for people of all sexual orientations and so we were not in a position to be able to exactly match the group sizes. Recruitment to this phase of the project stopped when we had a number that we deemed should yield over 20 participants per group.

The mean age of the sample was 25.5 years (SD = 8.5, range 18–69) though one participant did not give this information. The majority of the sample defined themselves as “white” (91.7%), with 4.1% as “black,” 2.7% as “Asian,” and 1.4% as “other.”

### Procedure

Before testing took place, participants were given a detailed information sheet that explained the nature of the experiments and questionnaires and that the data from the tasks would be kept confidentially. They were encouraged to ask questions about the tasks and procedures. They then signed a consent form. We then asked them to fill out the demographic questionnaire that included questions about how they described themselves in terms of their sexuality, the Kinsey scale (Kinsey, Pomeroy, & Martin, 1948), and a feeling thermometer about their sexual interests. Participants then completed a battery of tests that looked at different aspects of their sexuality and included both physiological recordings and behavioral tasks. Some of these have already been reported (Snowden et al., 2019). The sex IATs were always presented as the second set of tasks in this battery (following pupillometry measurements). Among the sex IATs, the gender-sex IAT was always presented first. The order of the other two sex IATs (women-neutral and men-neutral) was randomized. The priming task was then presented.

### Stimuli and Materials

#### Demographic Information

Participants were given a sheet with open-ended questions including “How do you describe your gender?” and “How do you describe your sexual orientation?” They were also asked to describe their ethnic group and their age.

#### Kinsey Scale

Sexual attraction was evaluated by a Kinsey scale with seven options. Option 0 was labeled as “Exclusively attracted to the other gender,” Option 3 was labeled as “Equally attracted to both genders,” and Option 6 was labeled as “Exclusively attracted to the same gender.” The seventh option was an “X” and was labeled “non-sexual or other.”

#### Feeling Thermometer

Direct ratings of feelings toward the construct pairs “sex with men” and “sex with women” were obtained using the feeling thermometer, which employs the heuristic of a thermometer. Participants rated feelings from “cold/unfavorable” at 0 to “warm/favorable” at 100 by circling the appropriate number on the scale.

#### Implicit Association Tests

The IAT requires the participant to categorize stimuli, via pressing one of two buttons, as they appear (one by one) on the computer screen running at 60 Hz. Stimuli and response were controlled by the DirectRT software. The screen was 48 cm wide and 30 cm in height. The images were presented in the center of the screen and were 25 cm wide and 15 cm high. The words were presented in the center of the screen and had a letter size of 1 cm. Labels (e.g., “men or sex”) were placed in the upper right and left of the screen to serve as a reminder of the correct responses. Participants sat approximately 57 cm from the screen and used the keyboard to give their responses.

We first describe the gender-sex IAT in detail and will then describe the changes made to produce the men-sex and the women-sex IATs. We represented the concept of “men” by the use of 8 pictures of men (all pictures were taken from the International Affective Picture System (IAPS: Lang, Bradley, & Cuthbert, 1997; IAPS Nos.: 4460, 4470, 4490, 4503, 4520, 4534, 4550, 4561) and the concept “women” with 8 pictures of women (IAPS Nos.: 4002, 4003, 4141, 4142, 4210, 4232, 4235, 4240). The pictures all depicted a single person either nude or partially dressed. We made an approximate attempt to match the pictures according to pose, ethnicity, etc., but no formal measurements were made.

The concepts of “sex” and “not sex” were represented by words that in pilot work (which included both offender and non-offender samples: Gray, Brown, MacCulloch, Smith, & Snowden, 2005) had been unanimously categorized as belonging to one of these categories. The sex words were: sex, fuck, lick, cum, cock, kiss, lust, and suck. The not-sex words were laugh, eye, toe, elbow, run, smile, walk, and knee.

The IAT contained two stages. In the first stage, participants classified pictures of men or sex words on the right button and pictures of women and not-sex words on the left button. Participants were instructed to “Try and respond as fast as you can, without making many errors.” Fifty-six trials (28 pictures and 28 words) were then presented in random order save that each word or picture was used at least once. Participants were then given a second set of instructions for stage 2. They were told that the response to the pictures was the same (right button for pictures of men, left for pictures of women) but that the response to the words had changed and they should now press the left button for sex words and right button for not-sex words. Fifty-six trials were then presented. For each block, the first eight trials were regarded as practice trials and were not analyzed.

The men-sex and women-sex IATs were as identical as possible to the gender-sex IAT. The major change was that one of the set of gender pictures was replaced by a set of neutral images chosen for their lack of any sexual connotation and low valence and low arousal ratings on the IAPS and included pictures of natural scenes and man-made objects (Nos.: 5220, 5260, 5300, 5390, 5660, 5875, 7000, 7020).

### Priming Task

The equipment and stimuli (pictures and words) were the same as used for the IATs. Each trial consisted of a fixation cross (1000 ms), the priming image (200 ms), and then the target word which remained until the participant responded. The participants completed 8 practice trials (all using a neutral prime not used in the data collection phase) followed by 120 trials (40 men primes, 40 women primes, 40 neutral primes) with 20 trials of each prime being followed by a sex word and 20 of each by a not-sex word. Trials were presented in random order that used a different seed for each participant.

#### Group Formation

For statistical analysis we formed three groups. The gynephilic group consisted of people who self-reported as being heterosexual and gave Kinsey ratings of 0 or 1. One participant who self-identified as heterosexual but gave a Kinsey rating of 2 and was removed from the analysis leaving *N* = 32. For the ambiphilic group, we included people who self-reported as being bisexual and had Kinsey ratings of 2–4. Two participants self-reported being bisexual but gave Kinsey ratings of 0 or 1, one reported being pansexual, and one reported being transgender. These data from these participants were not analyzed in order to increase the homogeneity of the group to cisgender ambiphilic men (*N* = 20). The androphilic group (*N* = 18) consisted of participants who self-identified as being homosexual and gave Kinsey ratings of 5 or 6.

### Data Reduction

Data from the IAT task are often analyzed by creating a D-score that combines both the RTs and errors and corrects for differences in overall speed by dividing by the standard deviation of responses (see Greenwald, Nosek, & Banaji, 2003). However, data from the prime task are not typically analyzed in this fashion. Hence, in order to be consistent across the studies in the present paper, our main analysis for all tasks consisted of an analysis of reaction times (see below). However, for the IAT tasks we also analyzed the data using D-scores in order to be consistent with the large body of IAT research. The D-scores analyses are available in the Supplementary Materials. We acknowledge that using two data analysis techniques increases the familywise error and this increases the chance of Type 1 (false positive) results. This was considered in the interpretation of the results. The pattern of results was highly similar using both data analysis techniques, but there was one instance (the comparison of ambiphilic and androphilic groups on the male-neutral IAT) where one technique (D-score) produced a significant result whereas the other (RTs) did not. To protect against a Type 1 error due to the inflated familywise error, we regarded this difference as not significant.

The data from the means from the IAT tended to show a non-normal distribution typical of reaction time tasks. These data were transformed by a reciprocal transform. The transformed data showed no departure from a normal distribution (Kolmogorov–Smirnov) and were used for the statistical analyses. However, the raw data are used for the figures and tables. The data from the prime task followed a normal distribution and formal tests (Kolmogorov–Smirnov) were not significant. Hence, these data were not transformed. For the explicit ratings, the data were bimodal and hence nonparametric statistics were used.

## Results

### Feeling Thermometer

One participant (ambiphilia group) did not want to complete the explicit rating task. For the feeling thermometer, gynephilic men gave more highly favorable ratings to sex with women than sex with men (97.2 vs. 5.6; Z= 5.14, *p* < .001) while androphilic men showed the opposite bias (13.1 vs. 95.8; Z= 3.66, *p* < .001). The ambiphilic men showed approximately equal favorability to sex with women and men (81.6 vs. 73.7; Z= 0.91, *p* = .36).

### Gender-Sex Implicit Association Test

Data from four participants (one gynephilic, two ambiphilic, and one androphilic) were removed due to excessive error rates (> 30%). The reaction time data were then screened for outliers (> 3 SD from mean), but none were found.

Reliability was assessed by calculating the difference score between the RTs for the men-sex block and the women-sex block for odd and even trials separately. The correlation between scores was then calculated, and the Spearman–Brown correction was applied. The resulting reliability coefficient was very high (*r* = .91, 95% CI [.86, .94], *p* < .001).

**Fig. 1:**
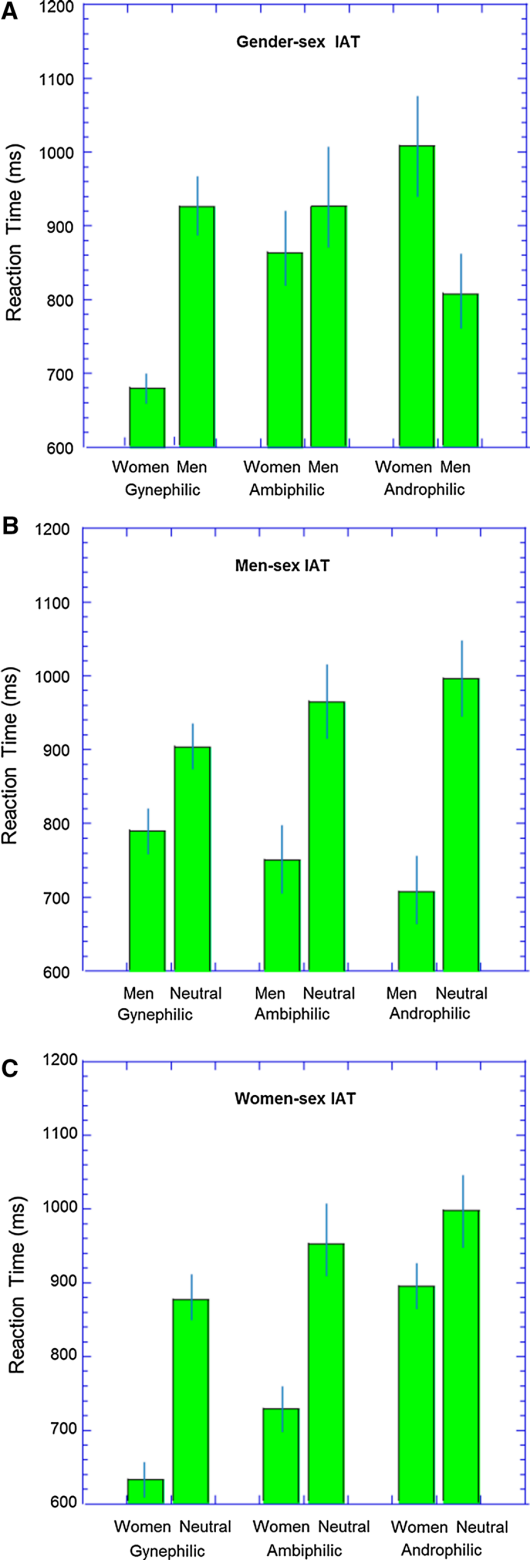
Results from the IAT experiments. Error bars represent ± 1 standard error of the mean (SEM). **a** Results from the gender-sex IAT, **b** results from the men-sex IAT, **c** results from the women-sex IAT

The results are illustrated in Fig. 1a. A two (target: men or women paired with sex) by three (group: gynephilic, ambiphilic or androphilic) ANOVA showed an effect of sexual target (*F*[1, 63] = 6.34, *p* = .014, *η*_p_^2^ = .091, 95% CI [.00, .24]) but not of group (*F*[2, 63] = 1.82, *p* = .17). The interaction was significant (*F*[2, 63] = 50.32, *p* < .001, *η*_p_^2^ = .62, 95% CI [.45, .71]).

Table 1 shows the planned comparisons. The gynephilic group showed faster performance for the women-sex condition, while the androphilic group showed the opposite result. As predicted, the data for ambiphilic men did not produce a significant IAT effect. However, it should be noted that this test had fairly low power and the confidence intervals allow for the possibility of a large bias toward faster performance on the women-sex condition.

**Table 1:** Results from the IAT experiments

	Gender-sex IAT	Men-sex IAT	Women-sex IAT
Gynephilic			
*n*	31	29	30
IAT effect (ms)	244.3**	116.8**	242.1**
Effect size	1.28 [0.88, 1.74]	.65 [0.28, 1.03]	1.68 [1.18, 2.27]
Ambiphilic			
*n*	18	17	18
IAT effect (ms)	62.0	217.1**	222.0**
Effect size	.22 [− 0.11, 0.57]	.94 [0.52, 1.45]	1.45 [0.81, 2.12]
Androphilic			
*n*	17	16	18
IAT effect (ms)	− 201.9**	291.8**	102.1*
Effect size	.76 [0.30, 1.28]	1.42 [0.88, 2.10]	.43 [0.08, 0.82]

The IAT effect size is the difference between the two conditions (in ms). The effect size is Hedge’s G, and the figures in square brackets are the 95% confidence intervals. For the gender-sex IAT, the data were coded so that the contrast was women–men so positive effects reflect faster RTs on the women-sex condition

* *p* < . 05; ** *p *< .01

We also compared the magnitude of the IAT across the three groups. The gynephilic group had a larger IAT score than the ambiphilic group (*t*[47] = 5.48, *p *< .001, *g* = 1.60, 95% CI [.95, 2.29]), and the ambiphilic group had a larger score than the androphilic group (*t*[33] = 4.03, *p *< .001, *g* = 1.33, 95% CI [.62, 2.09]).

This pattern of results could arise if the ambiphilic group was actually comprised of a mixture of men some with gynephilic-like responses and some with androphilic-like responses. If this were the case, then each individual on the gender-sex IAT should produce a strong difference response (between the men-sex and women-sex blocks). However, at a group level these large individual effects would cancel out in the ambiphilic group. We examined this idea by calculating a difference score between the men-sex and women-sex conditions. We then took the absolute value of the difference score for each person (ignoring the sign of the score) and plotted this against the Kinsey rating (for similar analyses, see Rieger & Savin-Williams, 2012). If individual ambiphilic men have similar associations to both men and women, we would expect these absolute scores to be lower for those with Kinsey scores near the middle of the Kinsey range.

**Fig. 2:**
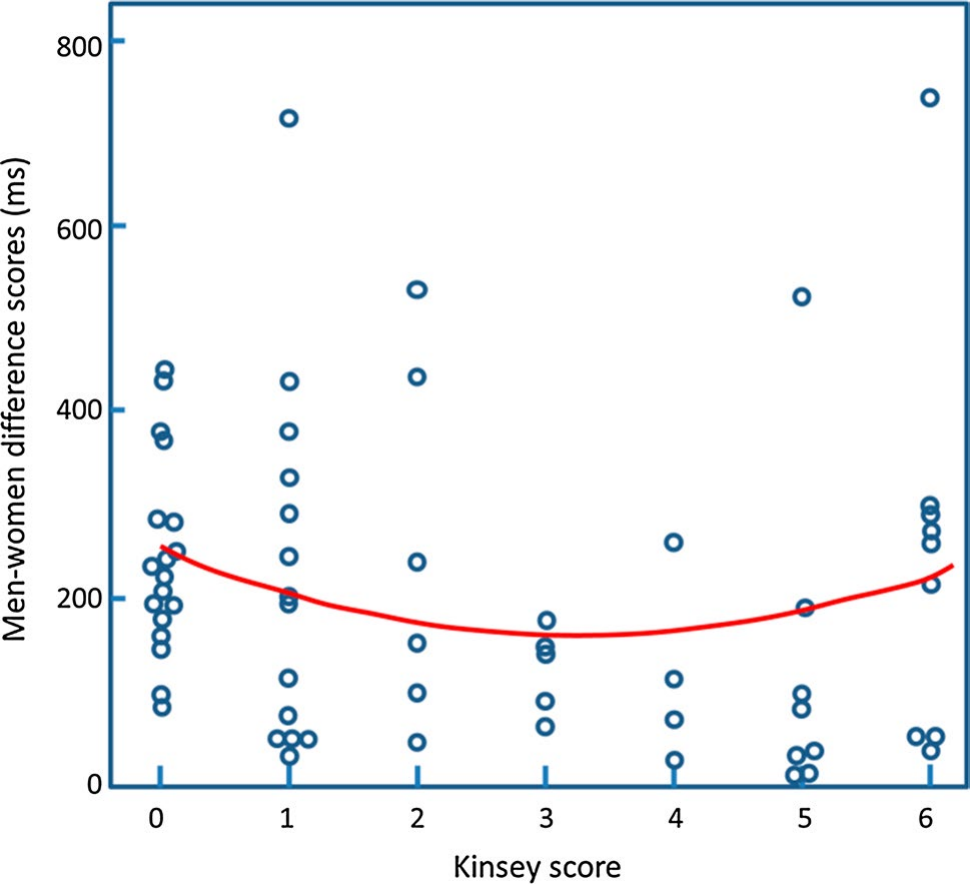
The absolute difference score between men-sex and women-sex blocks for the gender-sex IAT for each participant are plotted against their Kinsey rating. The curve is the best fitting quadratic function

The results are shown in Fig. 2. The data appear to show smaller absolute scores for the individuals with mid-range Kinsey scores. This was supported by statistical analysis that showed that the linear term did not produce a significant model (*R*^2^ = .004, *p* = .60), but that addition of the quadratic term produced a greater model fit (Δ*R*^2^ = .10, CI 95% [.01, .26], *p* = .01). Hence, this analysis suggests that the ambiphilic group is made up of (at least some) individuals with similar sexual associations to both men and women and not just by individuals with sexual associations to men and some individuals with sexual associations to women.

### Men-Sex Implicit Association Test

The data from three participants (one from each group) were lost due to equipment error. Four participants (one gynephilic, two ambiphilic, and one androphilic) were removed due to excessive error rates (> 30%). The reliability of the men-sex IAT was high (*r* = .73, 95% CI [.59 .83], *p* < .001).

The results are illustrated in Fig. 1b. A two-by-three ANOVA showed a significant effect of target (*F*[1, 60] = 112.07, *p* < .001, *η*_p_^2^ = .65, 95% CI [.50, .74]), no main effect of group (*F*[2, 60] = 0.08, *p* = .92, *η*_p_^2^ = .003), and a significant target-group interaction (*F*[2, 60] = 7.22, *p* = .002, *η*_p_^2^ = .19, 95% CI [.03, .34]).

Planned comparisons (see Table 1) showed that all three groups were faster under the men-sex condition that the neutral-sex condition. The crucial prediction was that the ambiphilic group would show a greater IAT effect than the gynephilic group. This was supported (*t*[45] = 2.27, *p *= .03, *g* = 0.68, 95% CI [.09, 1.29]). The difference between the ambiphilic group and the androphilic group was not significant (*t*[31] = 1.21, *p *=* .*23, *g* = 0.39, 95% CI [− 0.30, 1.08]).

### Women-Sex Implicit Association Test

Data from one participant (gynephilic) were lost due to equipment problems. Data from one gynephilic and two ambiphilic participants were excluded due to high error rates (> 30%). The reliability of the women-sex IAT was high (*r* = .80, 95% CI [.69 .87], *p* < .001).

The results are illustrated in Fig. 1c. A two-by-three ANOVA showed a significant effect of target (*F*[1, 63] = 122.33, *p* < .001, *η*_p_^2^ = .66, 95% CI [.51, .75]) and of group (*F*[2, 63] = 6.86, *p* = .002, *η*_p_^2^ = .18, 95% CI [.03, .33]), with a significant interaction (*F*[2, 63] = 12.89, *p* < .001, *η*_p_^2^ = .29 95% CI [.10, .44]).

Planned comparisons (see Table 1) showed that all groups were faster under the women-sex condition compared to the neutral-sex condition. The crucial prediction was that the ambiphilic group would show a greater IAT effect than the androphilic group. This was supported (*t*[34] = 3.12, *p *= .004, *g* = 1.30, 95% CI [.67, 1.96]). As expected, the difference between the ambiphilic group and the gynephilic group was not significant (*t*[46] = 1.82, *p *= .08, *g* = 0.53, 95% CI [− 0.06, 1.13]).

### Priming Task

Data from two participants (one gynephilic and one ambiphilic) were lost due to equipment problems, and four other data sets (two gynephilic and two ambiphilic) were removed due to high error rates (> 30%). Reliability was assessed by calculating the difference score between the RTs for the men-sex- and women-sex-related trials [(men_sex–women_sex) + (women_notsex–men_notsex)] for odd and even trials separately. The correlation between scores was then calculated, and the Spearman–Brown correction was applied. The resulting reliability coefficient was moderate (*r* = .57, 95% CI [.38 .72], *p* < .001).

**Fig. 3:**
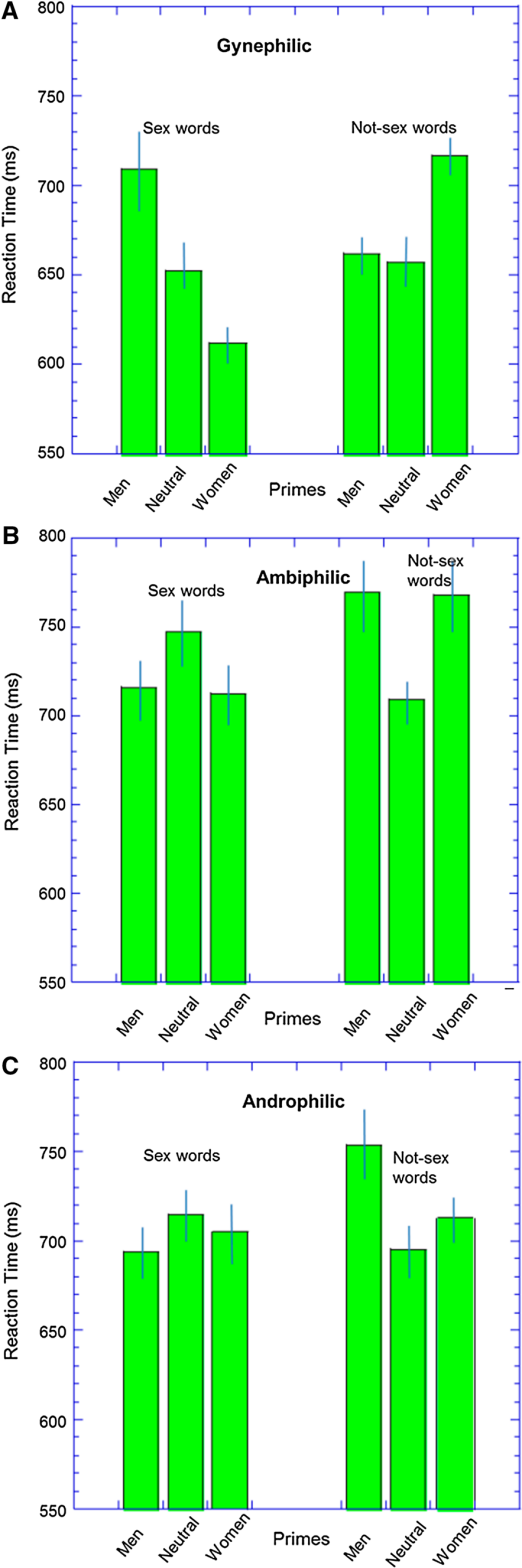
Results from the priming task. Error bars represent ± 1 standard error of the mean (SEM). **a** Results for the gynephilic group, **b** results for the ambiphilic group, **c** results for the androphilic group

The results are illustrated in Fig. 3. A two (word: sex vs. not-sex) by three (prime: pictures of men, women, or neutral) by three (group: gynephilic, ambiphilic or androphilic) ANOVA showed no significant main effects. However, there was a significant prime by target interaction (*F*[2, 120] = 9.82, *p* = .001, *η*_p_^2^ = .14, CI 95% [.04, .25]) and a three-way interaction, (*F*[4, 120] = 8.78, *p* < .001, *η*_p_^2^ = .23, CI 95% [.03, .23]).

To understand these complex interactions, we examined each group in turn using paired t-tests and we also calculated a combined priming score (priming for sex words–priming for not-sex words). The full set of priming effects, effect sizes, and confidence interval are given in Table 2. For the gynephilic group, all the priming effects were in the expected direction (e.g., the women primes reduce RTs for the sex words but increased RT for the not-sex words in comparison to the neutral primes) save for the lack of a significant difference between the men primes and neutral primes. For the androphilic men, this pattern of results was approximately the mirror image of those of the gynephilic men; however, the only significant priming effects were that men primes increased RTs for the not-sex words and produced a negative combined priming score. Crucially, the pattern of results was different for the ambiphilic men. The contrasts between men and women primes were all small (< 5 ms, *g*s < 0.05). However, both the men primes and the women primes increased RTs to the not-sex words. (There was also a trend for both primes to decrease RTs to the sex words, but this was not significant.) Using the combined priming score, there were clear effects of sexual priming for both gender primes.

**Table 2:** Results from the priming task

		*n*	Men versus women	Men versus neutral	Women versus neutral
Gynephilic		29			
Sex	RT effect		96.9**	57.0**	− 39.9*
	Effect size		1.07 [0.60, 1.52]	0.43 [0.17, 0.70]	0.33 [0.07, 0.61]
Not sex	RT effect		− 54.6**	5.2	59.8**
	Effect size		0.74 [0.32, 1.15]	0.04 [− 0.20, 0.29]	0.48 [0.23, 0.76]
Combined	RT effect		151.5**	51.8*	− 99.7**
	Effect size		1.04 [0.58, 1.49]	0.47 [0.08, 0.85]	0.93 [0.49, 1.36]
Ambiphilic		16			
Sex	RT effect		3.3	− 31.0	− 34.3
	Effect size		0.03 [− 0.46, 0.52]	0.17 [− 0.06, 0.40]	0.18 [− 0.11, 0.48]
Not sex	RT effect		.8	60.4*	59.5*
	Effect size		0.01 [− 0.48, 0.49]	0.34 [0.04, 0.66]	0.36 [0.01, 0.73]
Combined	RT effect		2.5	− 91.3*	− 93.8*
	Effect size		0.02 [− 0.47, 0.51]	0.68 [0.13, 1.22]	0.61 [0.07, 1.14]
Androphilic		18			
Sex	RT effect		− 10.8	− 20.9	− 10.2
	Effect size		0.12 [− 0.34, 0.58]	0.16 [− 0.13, 0.46]	0.08 [− 0.24, 0.40]
Not sex	RT effect		39.9	58.7**	18.8
	Effect size		0.41 [− 0.07, 0.89]	0.36 [0.09, 0.64]	0.12 [− 0.22, 0.46]
Combined	RT effect		− 50.7	− 79.6**	− 28.9
	Effect size		0.37 [− 0.11, 0.84]	0.78 [0.24, 1.30]	0.20 [− 0.26, 0.67]

RT effect is the difference between the two prime conditions (in ms), and the effect size is Hedge’s G with the 95% confidence intervals given in square brackets

* *p* < . 05; ** *p *< .01

**Fig. 4:**
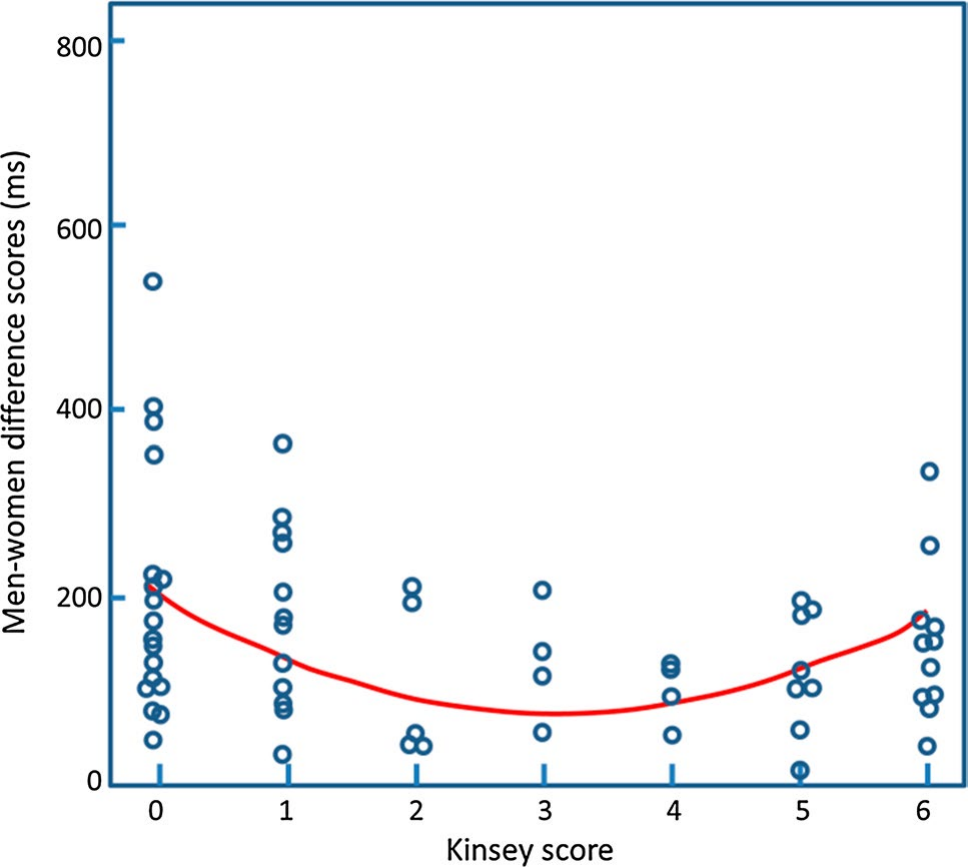
The absolute difference score between the men-sex (and women-not-sex) and women-sex (men-not-sex) trials for the priming task are plotted against Kinsey rating. The curve is the best fitting quadratic function

As for the IAT data, it might be argued that these results could possibly arise if the ambiphilic group was actually comprised of a mixture of men some of which had gynephilic-like responses and some with androphilic-like responses. Once again, we calculated the absolute difference score for the men vs women primes and plotted this against the Kinsey score (Fig. 4). The data appear to show smaller absolute scores for the individuals with mid-range Kinsey scores. This was supported by statistical analysis that showed that the linear term alone produced a significant model (*R*^2^ = .07, *p* = .03) but that addition of the quadratic term produced a greater model fit (Δ*R*^2^ = .07, CI 95% [.00, .22], *p* = .03). Hence, this analysis is consistent with ambiphilic sexual associations for those with Kinsey score in the center of the range.

### Supplementary Analysis

The correlations between all the measures of sexual interest are available in the Supplementary Materials (Table S1).

## Discussion

The major aim of this study was to examine whether men that identified as ambiphilic would show automatic sexual appraisals of images that differ from those of gynephilic or androphilic men in showing evidence for attraction to both men and women. In a gender-sex IAT the ambiphilic group did not show a significant overall IAT effect and their scores differed from both the gynephilic and androphilic groups. One possibility was that the ambiphilic group may have consisted of some individuals with gynephilic attractions and some with androphilic attractions. However, we found no evidence for this. On separate IATs that aimed to examine attraction to men and attraction to women in isolation, the ambiphilic men showed similar attraction to both men and women, whereas the gynephilic and androphilic men showed a greater effect for women and men, respectively. Finally, on a priming task the ambiphilic men showed priming by both images of men and women, whereas the gynephilic men were primed only by images of women and the androphilic men were only primed by images of men.

Before discussion of the implications of these results, we first would like to note some small differences between the predicted results and the actual results. The major differences are that for the women-sex IAT the androphilic men show greater sexual associations to images of women than to neutral images, and for the men-sex IAT the gynephilic men show greater sexual associations to images of men than to neutral images. We predicted neither association. These results seem to reflect that men have greater sexual associations to humans in general (even if they are of the non-preferred gender category) than neutral stimuli (such as natural and man-made scenes and objects).

As outlined earlier, the information processing model of Janssen et al. (2000) includes early automatic sexual appraisal of a stimulus (which we believe are being measured by the IAT and priming tasks) that give the stimulus emotional meaning. This early sexual response may lead to controlled attention processing of relevant sexual features and the further development of genital responses and the subjective feelings of sexual arousal.

Our results are supportive of recent results using pupil responses (Rieger & Savin-Williams, 2012) and genital responses (Rieger et al., 2015; Rosenthal et al., 2011) in amphiphilic men. However, not all experiments have found this result of approximately equal responses to men and women. Snowden et al. (2019) measured pupil dilation to briefly presented images of men and women in gynephilic, ambiphilic, and androphilic men and women. All groups showed greater dilation to the images of men (for a similar finding see Aboyoun & Dabbs, 1998), and this effect was greatest for gynephilic men and gynephilic women. They speculate that as pupil dilation reflects a general arousal response rather than a specific sexual arousal (Bradley, Miccoli, Escrig, & Lang, 2008) the dilation might have been caused by “novelty” or “shock” response to these images. It is most notable that the experiment of Rieger and Savin-Williams (2012) used videos lasting many seconds (and looked at pupillary responses across these long time periods) compared to the brief stimuli and response window used by Snowden et al. and Aboyoun and Dabbs. Pupillary responses to these longer duration stimuli might be reflecting more controlled or deliberate processes outlined in the information processing model compared to the more immediate reaction to the stimuli when it is presented briefly. However, we note there is increasing evidence from other experiments to suggest that the pupillary responses to sexual images are correlated with other measures of sexual interest such as the IAT (Ó Ciardha, Attard-Johnson, & Bindemann, 2018). Further work is needed to understand the conditions under which these automatic cognitive appraisals are consistent or inconsistent with this general measure of sympathetic nervous system arousal.

As discussed in the introduction, the findings from experiments using genital arousal have not always supported the notion of equal responses to men and women in ambiphilic men (e.g., Rieger et al., 2005; Tollison et al., 1979). It has been suggested that some experiments may have suffered from including some participants that were not genuinely ambiphilic. Rosenthal et al., therefore, used very stringent criteria for participant inclusion in their experiments (such as having been in a sexual relationship with at least two partners of each sex) and were able to show a clearer bisexual genital response in this population of ambiphilic men. Similar findings have been reported more recently (Rieger et al., 2013, 2015). It is notable that the stimuli used in experiments on genital responses tend to be of far greater duration (often minutes) that those used in the present experiments (< 1 s). It seems likely that the results from any experiment that uses long duration of stimulation are likely to reflect both the automatic processes and the controlled processes outlined in the information processing model (Janssen et al., 2000).

A large body of evidence has suggested that the sexual interests of men are category specific (in comparison to androphilic women who appear non-specific—see Chivers, 2017). Clearly, the present evidence is a challenge to this notion that all men are category specific in showing that ambiphilic men have approximately equal responses to both genders. At this point in time, there is no accepted model of how sexual attraction of any type is formed, although there appears to be evidence for both a role of genetics and of environment (Bailey et al., 2016). Without a model of how sexual interest develops, the reason why most men appear to form a category-specific sexual interest while others are less category-specific cannot be determined. We note the same dilemma appears for women where androphilic women appear to be non-categorical whereas those that identify as lesbian appear category-specific (Chivers, 2017). It would be of interest to examine female sexuality using similar indirect methods and we hope to report on such measurements in the future.

The present studies, and previous use of indirect methods to measure sexual interests (e.g., Rönspies et al., 2015), suggest that these techniques have utility to measures sexual interest. These techniques also have great pragmatic advantages over techniques such as genital arousal which require complex equipment and the presentation of sexual images, and are, of course, highly invasive. The fast and low-cost nature of these implicit tests make them ideal for epidemiological studies of populations (see Ciani & Battaglia, 2014) which in turn could be used to test models for the rates of various sexual interests in the population, including models of the expected rates of ambiphilia (Gavrilets & Rice, 2006).

### Limitations

Due to the relative rarity of ambiphilia and androphilia, and possible reluctance to volunteer for such experiments which ask intrusive questions about sexuality, the sample size used here was relatively small, but in line with previous experimental studies of ambiphilia in men. We were not able to perform more fine-grain analyses of the data, such as comparisons between people with more subtle differences in Kinsey score (e.g., 2 vs. 3 vs. 4) or those with a longer history of ambiphilic relationships. We were also reliant on people volunteering, and it may be that this produces a selection bias for people who are more willing to declare their non-monosexuality.

Given concerns about the social stigma that is faced by many with an ambiphilic orientation (Roberts, Horne, & Hoyt, 2015), there might be concerns that some might attempt to “fake” their responses on the IAT or prime tasks. However, to us this seems unlikely. First, all the participants were volunteers who were informed of the nature of our experiments (but not the details of the task) and so there does not seem to be any reason for them to be motivated to fake any aspect of their behavior or attitudes. Second, it is hard to fake the IAT without some knowledge of the task and some practice (Fiedler & Bluemke, 2005; Cvencek, Greenwald, Brown, Gray, & Snowden, 2010). Finally, any attempt to fake “not being ambiphilic” would run counter to the results we actually obtained where those in the ambiphilic group showed clear patterns of sexual attraction to both men and women on both the tasks we administered.

In our study, we simply used people’s self-reported sexual identity to form our groups. We acknowledge that such a division is crude and does not capture the diversity of plurisexual people (e.g., pansexual, queer, fluid, autogynephilia, gynandromorphophilia, etc.) and does not take account of changes in sexuality over time and/or context (Rosario, Schrimshaw, Hunter, & Braun, 2006). Further, we acknowledge that some people do not consider any label is appropriate to them. There is also evidence for subgroups within men that identify as ambiphilic that may have great importance in terms of sexual attraction (Rieger et al., 2013). Future research, using far larger samples, is needed if we are to understand the implicit sexual associations in these groups including those that are asexual.

### Conclusions

The data show that ambiphilic men have fast and automatic sexual appraisals of stimuli of both men and women. The methods used tap into early processes, are hard to fake or influence, and are often beyond the awareness of the individual. The techniques are also inexpensive and not invasive. We hope that a greater understanding of ambiphilia and other forms of sexuality or sexual attractions will increase greater acceptance of non-monosexuality and produce greater social acceptance of ambiphilic attractions and bisexual orientations (Mitchell, et al., 2015).

## Electronic supplementary material

Below is the link to the electronic supplementary material.

Supplementary material 1 (DOCX 15 kb)

Supplementary material 2 (DOCX 17 kb)
