# Supplementary material for: Sexual Attraction to Both Genders in Ambiphilic Men: Evidence from Implicit Cognitions
Source: Arch Sex Behav. 2019 Nov 5;49(2):503–15. doi: 10.1007/s10508-019-01552-6 (PMC7031175; doi:10.1007/s10508-019-01552-6)
Supplement: Supplementary file 1 — Supplementary material 1 (DOCX 15 kb) [file 10508_2019_1552_MOESM1_ESM.docx]

**Supplementary Materials I**

Data from the IATs were also analysed via the method similar to that recommended by Greenwald, Nosek, and Banaji (2003). Errors within each IAT-stage were penalised by substitution of error trial RTs with a score derived by adding a constant of 600 ms to the mean RT for that IAT-stage. The final IAT score (*D-*score) was then calculated as the difference between the mean RTs for the initial and reversed IAT-stage scores divided by the pooled standard deviation for both stages.

**Results**

**Gender-sex IAT.**

Gynephilic men showed a positive D-score indicative of sexual attraction to women (*M* = 0.65; 95% CI [0.49, 0.82]), while androphilic men had a negative D-score indicative of sexual attraction to men (*M* = -0.60; 95% CI [-0.81, -0.38]). The D-score for ambiphilic men was not significantly different from zero (*M* = 0.14; 95% CI [-0.05, 0.34]). A one-way ANOVA showed a significant effect of group, *F*(2, 68) = 43.04, *p* < .001) and all pairwise comparisons were significant (*p*s < .001).

**Men-sex IAT.**

All men showed a positive D-score indicative of sexual attraction to men rather than objects (gynephilic: *M* = 0.36; 95% CI [0.22, 0.50]; ambiphilic: *M* = 0.65; 95% CI [0.47, 0.82]; androphilic: *M* = 0.93; 95% CI [0.74, 1.12]). A one-way ANOVA showed a significant effect of group, *F*(2, 65) = 12.25, *p* < .001) and all pairwise comparisons were significant (*p*s < .05).

**Women-sex IAT.**

All men showed a positive D-score indicative of sexual attraction to women rather than objects (gynephilic: *M* = 0.81; 95% CI [0.66, 0.96]; ambiphilic: *M* = 0.60; 95% CI [0.41, 0.78]; androphilic: *M* = 0.32; 95% CI [0.12, 0.52]). A one-way ANOVA showed a significant effect of group, *F*(2, 67) = 17.76, *p* < .01) and all pairwise comparisons were significant (*p*s < .05).

**Reference.**

Greenwald, A. G., Nosek, B. A., & Banaji, M. R. (2003). Understanding and using the implicit association test: I. An improved scoring algorithm. *Journal of Personality and Social Psychology, 85*, 197-216.
