# Supplementary material for: Sexual Attraction to Both Genders in Ambiphilic Men: Evidence from Implicit Cognitions
Source: Arch Sex Behav. 2019 Nov 5;49(2):503–15. doi: 10.1007/s10508-019-01552-6 (PMC7031175; doi:10.1007/s10508-019-01552-6)
Supplement: Supplementary file 2 — Supplementary material 2 (DOCX 17 kb) [file 10508_2019_1552_MOESM2_ESM.docx]

Supplementary Materials II

Table S1. Correlations between the measures of sexual interest.

|  |  |  | 2 | 3 | 4 | 5 | 6 | 7 |
| --- | --- | --- | --- | --- | --- | --- | --- | --- |
| Explicit | 1 | Feeling Thermometer | .71** | -.27* | .27* | .37** | -.34** | .14 |
|  |  |  |  |  |  |  |  |  |
| IAT | 2 | Female v male | - | -.28* | .28* | .55** | -.33** | .31** |
|  | 3 | Male vs neutral |  | - | -.04 | -.17 | .02 | -.17 |
|  | 4 | Female vs neutral |  |  | - | -.03 | -.02 | .05 |
|  |  |  |  |  |  |  |  |  |
| Prime | 5 | Female v male |  |  |  | - | -.53** | .71** |
|  | 6 | Male vs neutral |  |  |  |  | - | -.23 |
|  | 7 | Female vs neutral |  |  |  |  |  | - |

** p* > .05*; **p* < .01
